# Attitude Toward Depression in Thai Physicians compared with general population

**DOI:** 10.1192/j.eurpsy.2023.754

**Published:** 2023-07-19

**Authors:** C. Charnsil

**Affiliations:** Chiangmai University, Chiangmai, Thailand

## Abstract

**Introduction:**

High stigma has been considered an important cause for the low rates of help-seeking, lack of access to care, undertreatment, material poverty, and social marginalization.Physicians commonly know about depression but are reluctant to seek mental health treatment.

**Objectives:**

This study aimed to examine the attitude toward depression in Thai physicians compared with the general population.

**Methods:**

A cross-sectional descriptive study was conducted on Thai physicians and the general population. We used the Depression Stigma Scale in the Thai version to assess stigma. The Depression stigma scale was distributed via the internet with a google form program.

**Results:**

Two thousand eighty-three participants responded to the questionnaire. Comparing the Depression Stigma Scale of the general population and physicians by using an independent test demonstrated that there was a significant difference between the two groups (p < 0.001) with an average total score of physicians higher than the general population (37.47 and 35.73, respectively). There was a significant difference in the Perceived Stigma Subscale in the general population p < 0.001 and physicians but not in the Personal Stigma Subscale. A significant difference was shown between the Personal Stigma Subscale of male and female physicians (P < 0.05). No significant difference was demonstrated between the Perceived Stigma Subscale of male and female physicians. However, the male and female general population had no significant differences in the Depression Stigma Scale.

**Conclusions:**

Physicians had higher depression stigma than the general population, especially in perceived stigma.

**Disclosure of Interest:**

None Declared

